# Oxytocin levels in low-risk primiparas following breast stimulation for spontaneous onset of labor: a quasi-experimental study

**DOI:** 10.1186/s12884-019-2504-3

**Published:** 2019-10-12

**Authors:** Kaori Takahata, Shigeko Horiuchi, Yuriko Tadokoro, Erika Sawano, Kazuyuki Shinohara

**Affiliations:** 1 Graduate School of Nursing Science, St. Luke’s International University, 10-1 Akashi-cho, Chuo-ku, Tokyo, 104-0044 Japan; 2 St. Luke’s Maternity Care Home, 24 Akashi-cho, Chuo-ku, Tokyo, 104-0044 Japan; 3 Tokyo Healthcare University, 1-1042-2 Kaijincho nishi, Funabashi-shi, Chiba, 273-8710 Japan; 4 Graduate School of Biomedical Sciences, Nagasaki University, 1-12-4 Sakamoto, Nagasaki-shi, Nagasaki, 852-8523 Japan

**Keywords:** Breast stimulation, Salivary oxytocin, Induction of labor, Labor onset, Single nucleotide polymorphism, Pregnant women, Complementary therapies

## Abstract

**Background:**

Breast stimulation is performed to self-induce labor. However, there are apparently no reports on hormonal evaluation during stimulation for consecutive days in relation to induction effect. We evaluated the salivary oxytocin level following 3 consecutive days of own breast stimulation for 1 h each day compared with no breast stimulation.

**Methods:**

We used a quasi-experimental design. The participants were low-risk primiparas between 38 and 39 gestational weeks. Eight saliva samples per participant were collected at preintervention and 30, 60, and 75 min postintervention on the first and third days. The primary outcome was change in the salivary oxytocin level on the third day after 3 consecutive days of breast stimulation for 1 h each day compared with no breast stimulation. The secondary outcomes were the rate of spontaneous labor onset and negative events including uterine hyperstimulation and abnormal fetal heart rate.

**Results:**

Between February and September 2016, 42 women were enrolled into the intervention group (*n* = 22) or control group (*n* = 20). As there were differences in the basal oxytocin levels between the 2 groups, to estimate the change in the oxytocin level from baseline, we used a linear mixed model with a first-order autoregressive (AR1) covariance structure. The dependent variable was change in the oxytocin level from baseline. The independent variables were gestational weeks on the first day of intervention, age, education, rs53576 and rs2254298, group, time point, and interaction of group and time. After Bonferroni correction, the estimated change in the mean oxytocin level at 30 min on the third day was significantly higher in the intervention group (*M* = 20.2 pg/mL, *SE* = 26.2) than in the control group (*M* = − 44.4 pg/mL, *SE* = 27.3; *p* = 0.018). There was no significant difference in the rate of spontaneous labor onset. Although there were no adverse events during delivery, uterine tachysystole occurred in 1 case during the intervention.

**Conclusions:**

The estimated change in the mean oxytocin level was significantly higher 30 min after breast stimulation on the third day. Thus, consecutive breast stimulation increased the salivary oxytocin level. Repeated stimulations likely increase the oxytocin level.

**Trial registration:**

UMIN000020797 (University Hospital Medical Information Network; Prospective trial registered: January 29, 2016).

## Background

Many pregnant women exert conscious effort to induce labor using various methods such as breast stimulation before synthetic oxytocin infusion becomes necessary [1–3]. Breast stimulation is a natural method that requires no cost or technology, and the procedure can be taught by midwives and performed by pregnant women at their own convenience. A systematic review previously reported that breast stimulation for labor induction reduced the number of women who were not in labor after 72 h [4]. In a recent pilot randomized control trial (RCT), it was clarified whether breast stimulation aided the spontaneous onset of labor and vaginal delivery in 200 Indian pregnant women at term pregnancy [5]. Regarding the breast stimulation time and period, previous studies reported 60 min once a day for 3 days (minimum stimulation time and period) [6], more than 90 min twice a day for 3 days [7], 60 min three times a day for 3 days [8], 15–30 min twice a day for 1 week [5], and 1 h a day (encouraged 3 h or more) for 1 week or until born [9]. Other studies reported that breast stimulation instructions were shown in a movie for 2 min [6] or breast stimulation was initially performed in the hospital [8]. Most studies involved stimulation methods that were not strictly controlled interventions being mainly applied at home. What was common among these studies was stimulation of the nipple and areola for long hours by self-massage at home. However, there is still no standard definition of breast stimulation, thus the method, timing, time, and period vary from study to study. Moreover, an effective intervention time for breast stimulation has yet to be established. Additionally unknown are whether physiological alterations such as hormonal changes on consecutive days have a labor induction effect.

Breast stimulation promotes the production and release of oxytocin, which is considered to cause uterine contraction leading to spontaneous labor. Previous studies have measured the oxytocin level in pregnant women during a short breast stimulation time at various minute intervals in 1 day. The increase in the oxytocin level in pregnant women as induced by breast stimulation was either not significant [10, 11] or significant [12–14]. These vividly show the inconsistencies of the reported oxytocin levels induced by breast stimulation. The maximum breast stimulation times in previous reports were 12 [10, 12, 14], 30 [13], and 40 [11] minutes. What remains lacking are studies investigating the effects of breast stimulation for several days on changes in the oxytocin level. Moreover, there is a gap in knowledge related to repeat efficacious breast stimulation, oxytocin level, and spontaneous onset of labor.

Our previous feasibility study suggested the possible increase in salivary oxytocin level by breast stimulation on the third day of intervention [15]. Sixteen low-risk women in their late pregnancy participated in our previous study. They performed breast stimulation for 1 h each day for 3 days. Our previous study, however, lacked a control group. We also had to improve the saliva collection method because half of the collected samples had insufficient amount for saliva assay.

The present study aimed to evaluate the salivary oxytocin level after 3 consecutive days of breast stimulation for 1 h each day compared with no breast stimulation. The primary outcome was the change in oxytocin level on the third day. The secondary outcomes were the rate of spontaneous labor onset and negative events including uterine hyperstimulation and abnormal fetal heart rate.

## Methods

### Study design and participants

The present study used a quasi-experimental design with a control group. Data were collected from February 2016 to September 2016 in a single maternity hospital located in an urban area in Kanagawa, Japan. This is the same hospital as in our preliminary study [15].

The eligibility criteria for participation were as follows: (1) between 20 and 39 years of age, (2) having singleton and cephalic position and planning spontaneous delivery as low-risk pregnancy, (3) between 38 and 39 weeks of gestation, and (4) Asian and can read and write Japanese. Women were excluded from the study if they have (1) ongoing medications related to their gestation, (2) medical or pregnancy complications, (3) mental illness, (4) a medical history of assisted reproductive technology treatment, (5) a BMI above 25 before pregnancy, (6) planned on having an induced labor, or (7) already conducted breast stimulation for more than 10 min per day.

When eligible women at 34 weeks of gestation visited the hospital for a health check-up, they were recruited and they provided written informed consent. The participants were nonrandomly assigned into 2 groups. The first half was assigned to the control group and the last half to the intervention group. After participating in the study, the participants received monetary compensation by bank transfer (¥2000 or about $20 per day) based on the number of days participating in the intervention.

The warranted sample size was 15 subjects for each group in reference to previous reports on plasma oxytocin level in relation to short breast stimulation in pregnant women requiring 10–20 people [10–14]. Considering a dropout rate of 30% from the lack of saliva for assay using the improved saliva collection protocol from our previous study [15], the sample size needed was 22 women for each group.

All the participants and healthcare providers at the study settings and a biochemist who assessed the laboratory outcomes were masked regarding the participant allocations.

### Procedures

The intervention period was 3 consecutive days. Women visited a quiet room at the hospital after eating lunch on the first and third intervention days. The participants sat in a semi-Fowler’s position in a chair and were free to change their posture.

On the first day in the intervention group after the basal saliva collection, the women received an explanation of the breast stimulation method. The researcher or research assistant demonstrated the procedure for about 5 min to standardize the breast stimulation procedure as follows: (1) *rhythm*, confirmation of 69 beats per minute using a lighting sign of metronome in mute; (2) *overall posture*, explanation regarding the proper positioning of the arm or fingers for stimulation; (3) *finger position*, indication of the proper position on a breast model; (4) *pressure*, indication using a pressure-measuring instrument (Perineometer, OWOMED, Gyeonggido, South Korea) (the recommended stimulation pressure was < 10 mmHg, which is gentle and does not damage the nipple); (5) *final demonstration*, stimulation of the participant’s breast for about 5 counts according to the stimulation rhythm.

Each breast was stimulated for 15 min on each side and alternating for a total of 1 h per day, as simultaneous stimulation might cause uterine tachysystole [6, 7]. The nipple was stimulated directly by the participant using either the left or right hand. Pure lanolin nipple cream was used for a more comfortable stimulation [5, 9]. The cream was given to the participants who could apply it liberally. As an example of the breast stimulation procedure using the right hand, stimulation was performed by pinching only the nipple using the thumb and forefinger. The thumb position was in the “12 or 9 o’clock” direction of the nipple, with the forefinger placed opposite of the thumb.

On the second day, breast stimulation was performed at home before noon so that the participants can have access to treatment within hospital hours in case of emergency. All the necessary materials for breast stimulation were lent to the women. To confirm compliance of the participants and their safety, the women sent emails to researcher 10 min before the breast stimulation and within 10 min after the intervention indicating whether they had vaginal bleeding, premature rapture of membrane, or less fetal movement.

On the third day, the same procedure on the first day was performed at the hospital.

In the control group, on the first and third days after the first saliva collection, the women continued to watch a silent train movie. On the second day, they resumed their usual life activities. On the third day, they repeated the stimulation procedure performed on the first day.

### Outcome measurements

The primary outcome was a higher oxytocin level at 30 min on the third day in the intervention group than in the control group. All saliva samples on the first and third days were collected from 12:30 to 16:00 to control for diurnal effects [16, 17]. Saliva samples (*n* = 4) were collected before the intervention and at 30, 60, and 75 min after the start of the breast stimulation on the first and third days. This means that the samples were collected at the 30, 60, and 75 min time frame from the onset of breast stimulation. The total number of samples for each participant was 8. The participants were given the following instructions to avoid interference with saliva oxytocin assay [18]: (1) exercise or alcohol is prohibited on the day before the intervention; (2) caffeine intake is not allowed on the day of intervention; (3) smoking, brushing, eating, and drinking (except drinking water) are prohibited 1 h before saliva collection; (4) lipstick is not allowed.

Saliva collection was started after gargling [18]. Basal saliva oxytocin level was determined after 10 min of rest time while watching the movie “The World by Train” without the narration (silent movie). Both the intervention group and the control group watched the same movie which is familiar to the Japanese population. The participants were instructed to wait for 3 min for the saliva to accumulate naturally in their mouth. Then, saliva samples were collected in pre-chilled 2.0 mL polypropylene tubes (Eppendorf, NY, USA) that were subsequently stored on ice using a cut straw (passive drool). This process was repeated 3 times. The target amount of saliva to be collected was 2.0 mL [15, 19]. If the amount of saliva collected was small, the participants were instructed to self-massage their submandibular gland line.

After saliva collection, the samples were immediately stored in a freezer at − 80 °C. Oxytocin level was assayed using the method of Carter et al. (2007) [20]. We added the protease inhibitor aprotinin (500 KIU/mL) to inhibit metabolic breakdown of the peptide after thawing the saliva [21]. Oxytocin level was determined by enzyme-linked immunosorbent assay (ENZO Life Sciences, NY, USA) (http://static.enzolifesciences.com/fileadmin/files/manual/ADI-900-153A_insert.pdf). The technical report by Salimetrics LCC stated that the acceptable intra-assay and inter-assay coefficients of variation are < 10 and < 15%, respectively. In the present study, the intra-assay and inter-assay coefficients of variation from the dilutions of test samples were < 2.68 and < 5.49%, respectively. Where possible, all samples were run in duplicate (assay rate: 81.7%), but some samples had insufficient volume of saliva and were run as a single assay. There were also samples that contained less saliva or more mucin and did not reach the amount of saliva required for a single assay. These cases were treated as missing data.

For the secondary outcome, a higher rate of spontaneous labor onset was found in the intervention group than in the control group. Data on labor onset were obtained from the medical records. Moreover, data on labor induction, labor augmentation, gestational weeks at delivery, mode of delivery, and labor duration were gathered.

For another secondary outcome, there was a difference in the basal oxytocin level between the oxytocin receptor gene SNPs. Recently, it has been reported that specific oxytocin receptor gene SNP has decreased oxytocin sensitivity. It has also been confirmed that in the GG type of rs53576, the duration of labor at the first stage is prolonged [22]. In addition, it has been found that the GG type of rs2254298 is related to a significantly low plasma oxytocin level [23]. Buccal mucosa samples were obtained using a sample collection swab (Epicentre, Wisconsin, USA). All the samples were analyzed for the presence of 2 oxytocin receptor gene polymorphisms (i.e., rs53576 and rs2254298) by genotyping using the TaqMan SNP assay.

Negative events included uterine hyperstimulation, abnormal fetal heart rate, premature rupture of membranes, weak uterine contractions, Apgar score (1 and 5 min), meconium stained liquor, neonatal intensive care unit admission immediately after birth, and stillbirth. Cardiotocography was used to record the FHR and uterine activity 30 min before stimulation and until 30 min after intervention.

### Statistical analyses

The baseline characteristics and outcomes were compared between the intervention group and the control group using an independent *t*-test, the chi-square test, or Fisher’s exact test. In Table 2, we used a linear mixed model with a first-order autoregressive (AR1) covariance structure to estimate the change in the oxytocin level from baseline. The dependent variable was change in the oxytocin level from baseline. The independent variables were gestational weeks on the first day of intervention, age, education, rs53576 and rs2254298, group, time point, interaction of group and time. All independent variables were treated as fixed effects. Bonferroni correction was performed to avoid multiple comparison problems. SNPs were divided into groups GG and AA/AG, and an independent *t*-test was performed for baseline oxytocin level analyses. Statistical analyses were performed using SPSS Statistics version 24.0 J (IBM Corp.).

### Ethical consideration

The study was approved by the Institutional Review Board of St. Luke’s International University, Tokyo, Japan (No. 15–085). This study was registered prospective in the Clinical Trials Registry of the University Hospital Medical Information Network in Japan (UMIN 000020797). Participation in this study was determined by voluntary intention. Even after giving consent, the participants could withdraw at any point in time. The participants were assured that they will not be placed at a disadvantage for non-participation in the research. The women who took part in this study provided written informed consent for participation.

## Results

### Retention rates and demographic characteristics

**Fig. 1:**
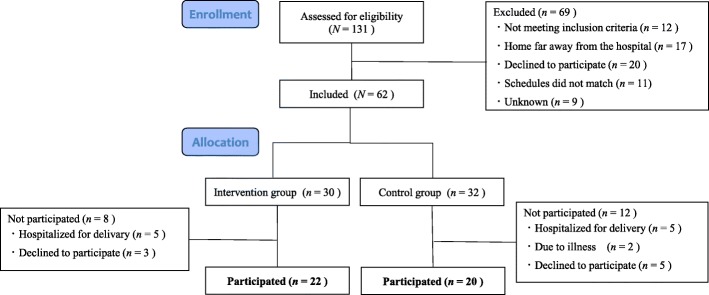
Flow diagram of participants. N: number of participants; n: number of subjects allocated to each group. The participants were not randomly placed into 2 groups. The first half of the participants was assigned to the control group and completed the intervention first. The last half was assigned to the intervention group and completed the intervention after the control group had finished

Of the 131 women assessed using the eligibility criteria, 62 women consented to participate in this research. In the control group, 32 women consented to participate, and 20 women started the intervention (no breast stimulation). In the intervention group, of the 30 women who consented to participate, 22 women started the intervention (breast stimulation). Thus, a total of 42 low-risk primiparas participated (Fig. 1). There were no significant differences in the baseline characteristics of the participants between the 2 groups (Table 1). There were also no correlations between the characteristics and the oxytocin baseline levels on the first day.

**Table 1:** Baseline characteristics of the study participants (*N* = 42)

	Intervention		Control		*p*-value
	(*n* = 22)		(*n* = 20)		
	*n*	%	*n*	%	
Age (years)	28.8	[3.8]	29.3	[3.7]	0.714
Gestational weeks	38.8	[0.4]	38.8	[0.5]	0.955
Living with partner	22	(100)	20	(100)	1.000
Married	22	(100)	20	(100)	1.000
Education ≥13 years	18	(81.8)	17	(85.0)	1.000
Due date confirmed by USG ≤ 12 gw	21	(95.5)	19	(95.0)	1.000
Fetal gender (boy)	12	(54.5)	13	(65.0)	0.491
Had contact with children	5	(22.7)	4	(20.0)	1.000
Taking medicine	6	(27.3)	5	(25.0)	0.867
Oral injury	1	(4.5)	0	0.0	1.000
Oxytocin receptor polymorphisms					
rs53576 (GG)	2	(9.5)	2	(11.8)	1.000
rs2254298 (GG)	10	(47.6)	8	(47.0)	0.976

*p*-value: using the two-tailed *t*-test, χ2 test, or Fisher’s exact test of comparisons between the intervention group and the control group

[ ] shows standard deviation; ( ) shows %

*USG* Ultrasonography

### Salivary oxytocin

The oxytocin level was significantly higher in the intervention group (*n* = 21, *M* = 159.6, *SD* = 101.7) than in the control group (*n* = 17, *M* = 91.8, *SD* = 39.5) at baseline on the first day (*p* = 0.009) (Table 2).

**Table 2:** Details of the comparison of the mean salivary oxytocin levels (pg/mL) between the intervention and control groups (*N* = 42)

		Intervention			Control			*p*-value
		*n*	*Mean*	*SD*	*n*	*Mean*	*SD*	
First day	Base	21	159.6	101.7	17	91.8	39.5	0.009
	30 min	19	165.4	130.0	17	78.3	33.9	0.010
	60 min	19	132.1	85.9	17	75.0	35.1	0.013
	75 min	20	147.6	89.2	16	67.1	33.2	0.001
Third day	Base	15	163.8	122.2	13	79.9	42.5	0.023
	30 min	14	168.5	110.2	13	76.0	26.5	0.008
	60 min	14	123.3	100.7	13	74.5	33.1	0.106
	75 min	16	127.0	99.4	13	72.7	25.7	0.050

An independent t-test was performed for oxytocin level analyses. Welch’s t-test was conducted when the data had unequal variances. In this table, the assumption of normality was adopted in 9 out of 16 samples. The assumption of normality was possible at baseline and at 75 min on the first day and at 30 min on the third day in both groups

*p*-value: using the two-tailed *t*-test

*SD* Standard deviation

The estimated change in the mean oxytocin level at 30 min on the third day was significantly higher in the intervention group (*M* = 20.2 pg/mL, *SE* = 26.2) than in the control group (*M* = − 44.4 pg/mL, *SE* = 27.3; *p* = 0.018) (Table 3). At other points, there were no significant differences.

**Table 3:** Estimated changes in the mean oxytocin level in the intervention and control groups using the linear mixed model with the baseline oxytocin level on the first day and the characteristic factors as covariates (*N* = 37)

		Intervention		Control		*p* ^*a*^	*p* ^*b*^
		*M*	*SE*	*M*	*SE*		
First	Base	*ref*		*ref*			
	30 min	6.1	25.3	−38.8	28.2	0.053	0.369
	60 min	−23.7	24.0	−42.1	25.1	0.169	1.000
	75 min	−8.6	24.9	−49.1	25.8	0.012	0.083
Third	Base	18.7	27.4	−40.1	29.1	0.021	0.144
	30 min	20.2	26.2	−44.4	27.3	0.003	0.018
	60 min	−19.7	25.9	−45.9	27.0	0.171	1.000
	75 min	−19.6	25.7	−49.1	26.9	0.119	0.832

The covariate was evaluated based on the basal oxytocin level on the first day = 121.1, gestational weeks on the first day of intervention = 38.7, age = 29.0

*M* Mean estimate level, *SE* Standard error, *ref* reference

^a^: Without correction, ^b^: With Bonferroni correction

Although there was no significant difference between the 2 groups, the estimated change in the oxytocin level at 60 min after the start of the intervention was lower than the baseline level in the intervention group on each day (first day, intervention group *M* = − 23.7, control group *M* = − 42.1, *p* = 1.000; third day, intervention group *M* = − 19.7, control group *M* = − 45.9, *p* = 1.000) (Table 3).

### Spontaneous onset of labor

There was no significant difference in the rate of spontaneous onset of labor between 18 women (81.8%) in the intervention group and 16 women (80.0%) in the control group (Table 4). There were no significant differences in the other delivery outcomes between the 2 groups.

**Table 4:** Delivery outcomes and negative effects (*N* = 42)

	Intervention		Control		*p*-value
	(*n* = 22)		(*n* = 20)		
	*n*	%	*n*	%	
Gestational weeks at delivery	40.0	[0.7]	40.0	[0.7]	0.771
Spontaneous onset of labor	18	(81.8)	16	(80.0)	1.000
Induced labor	4	(18.2)	4	(20.0)	
Onset of labor within 72 h after and within intervention	5	(22.7)	6	(30.0)	0.734
Mode of delivery					
Vaginal delivery	20	(90.9)	16	(80.0)	0.489
Instrumental delivery	2	(9.1)	3	(15.0)	
Cesarean section	0	(0.0)	1	(5.0)	
Weak uterine contractions	5	(22.7)	8	(40.0)	0.320
Labor augmentation	3	(13.6)	7	(35.0)	0.214
No synthetic oxytocin used during labor	7	(31.8)	11	(55.0)	0.212
Duration from intervention to delivery in patients who completed 3 days without induction of labor (Intervention, *n* = 13; Control, *n* = 12).					
First stage (minutes)	734	[497.1]	816	[663.9]	0.650
Second stage (minutes)	118	[179.0]	153	[241.4]	0.599
Third stage (minutes)	4	[2.9]	3	[1.4]	0.052
Duration of labor (hours)	14.2	[8.6]	16.9	[12.4]	0.441
Premature rupture of membranes	5	(22.7)	3	(15.0)	0.700
Uterine hyperstimulation	0	(0.0)	0	(0.0)	–
Bleeding					
Within the 2 h after birth (g): *M* [*SD*]	47	[22.2]	60	[39.4]	0.208
Total bleeding volume (g): *M* [*SD*]	317	[184.1]	450	[270.2]	0.074
Postpartum hemorrhage	3	(13.6)	7	(35.0)	0.152
Apgar Score at 1 min (< 7)	0	(0.0)	1	(5.0)	0.476
Apgar Score at 5 min (< 7)	0	(0.0)	0	(0.0)	–
Birth weight (in grams)	3090	[287]	3077	[277]	0.881
NICU admission immediately after birth	1	(4.5)	1	(5.0)	1.000
Meconium-stained liquor (≥ 2+)	1	(4.5)	4	(20.0)	0.174
Stillbirth	0	(0.0)	0	(0.0)	–

*p*-value: using the two-tailed *t*-test, χ^2^ test, or Fisher’s exact test of comparisons between the intervention group and the control group.[ ] shows standard deviation; ( ) shows %

*NICU* Neonatal Intensive Care Unit

### Polymorphisms of oxytocin receptor

All samples could be analyzed by SNP detection; the baseline oxytocin level could be analyzed on the first day in 21 women in the intervention group and in 17 women in the control group. However, there was also no significant difference between GG and AG/AA of rs2254298 and rs53576 and the baseline oxytocin level.

### Adverse events

During delivery, there was no significant difference in the adverse events between the 2 groups (Table 4).

During the intervention in the breast stimulation group on the first day, only 1 woman interrupted the intervention because of a painless uterine tachysystole with prolonged FHR deceleration. From 2 min after the interruption, severe variable deceleration within 3 min at the lowest point of 60 bpm was confirmed. After 30 min of monitoring, the obstetrician allowed the resumption of the experiment. However, because of the time schedule, the experiment was not resumed. The woman showed no abnormal features afterwards. Labor onset started 4 days after the intervention, and normal delivery occurred in 4.5 h.

On the second day of home intervention in the intervention group, there was no emergency response incident.

## Discussion

### Primary outcome: salivary oxytocin level

To the best of our knowledge, this is the first quasi-experimental study to clarify the changes in oxytocin levels as induced by breast stimulation over a 3-day period. At 30 min after the start of the intervention on the third day, the estimated change in the mean salivary oxytocin level was significantly higher in the intervention group than in the control group after adjustment of the baseline level on the first intervention day using a linear mixed model (*p* = 0.018, Table 3). At 30 min after the start of the intervention on the first day, the estimated mean salivary oxytocin level was not significantly higher in the intervention group than in the control group (*p* = 0.369**,** Table 3). It is possible that the oxytocin level in the intervention group was increased by the repeated breast stimulation. In previous studies, breast stimulation was performed only for 1 day, thus there is a possibility of showing inconsistent results [10–14]. There is also a possibility that adaptive changes occur by repeated exposure to endogenous oxytocin [24].

Although there was no significant difference, another noteworthy fact is that the oxytocin level at 60 min after the start of the intervention was lower than the baseline level in the intervention group (first day, *M* = − 23.7; third day, *M* = − 19.7). As breast stimulation was continued for 60 min, it was anticipated that the 60-min time point would indicate the peak of the oxytocin level; however, the oxytocin level decreased even when the breast stimulation was continued. As the oxytocin level gradually decreased in the control group, it is possible that the oxytocin level in the intervention group naturally decreased after reaching the peak level.

Several studies have recommended breast stimulation for more than 180 min per day [7–9]. A previous study reported that for women who delivered in less than 42 weeks of gestation, breast stimulation for 3 h or longer resulted in significantly shorter days to delivery than stimulation for less than 3 h [9]. However, the breast stimulation was performed at home and was not a strictly controlled intervention. On the other hand, our results suggested that breast stimulation may not increase the oxytocin level even if the breast is stimulated for more than 60 min. Earlier studies used longer intervention periods such as 7 days [5]. Importantly, it may be possible to increase the basal oxytocin level using breast stimulation by repeated interventions rather than by a long stimulation period.

### Secondary outcomes

There was no significant difference in the rate of spontaneous onset of labor in the 2 groups. Moreover, there was no significant difference in the rate of labor onset within 72 h after the intervention. A possible reason why this hypothesis is not supported aside from the sample size is the gestational weeks when the intervention was started. For women in their late gestational weeks, oxytocin receptor mRNA protein levels have been found to increase [25, 26]. It has also been shown that an increase in oxytocin levels and the number of oxytocin receptors are correlated with an increase in response to oxytocin [27] and a better response to synthetic oxytocin [28]. In previous RCTs, the number of gestational weeks when the breast stimulation intervention was started varied, namely, at the completion of 37 weeks [8], 38 weeks [5, 7], and 39 weeks [6, 9]. Thus, the present study cannot be considered as an earlier intervention compared with these previous studies.

In the present study, SNPs rs2254298 and rs53576 appeared to have not affected the basal oxytocin level; however, this outcome should be confirmed by re-examination with an appropriate sample size.

There were no adverse events during delivery similarly to previous studies [6, 7, 9]. During the experiment, 1 woman confirmed painless uterine tachysystole. FHR tracing shows the acid-base status of the fetus at the time of observation. Prolonged deceleration is classified as Category II FHR tracings. In this category, the tracings are insufficient to judge as predictive of an abnormal acid-base status and are observed daily in routine practice [29]. In the present study, all the participants were low-risk pregnant women who received regular prenatal check-up and had cardiotocography for FHR monitoring at least 30 min before the intervention. In previous studies, uterine hyperstimulation or tachysystole as an adverse event during the experiment was not reported. However, there was only 2 studies [7, 30] that confirmed FHR monitoring. In the future, it is recommended to perform daily fetal movement counts at home and the breast stimulation stress test in questionable cases before the intervention [9]. As described, it is necessary to pay sufficient attention to safety particularly for those undergoing intervention for the first time.

### Limitations of the study and suggestions for future studies

There are several limitations in this quasi-experimental study. *First*, the oxytocin levels were significantly higher in the intervention group than in the control group at baseline on the first day. The baseline differences were, however, unlikely to be affected by the storage period in the freezer. This is because saliva samples were collected, they were sent frozen for analysis and were analyzed once a month. The freezer storage time of the saliva samples from the control and intervention groups was almost the same, thus the intervention arm samples were not in the freezer for a shorter amount of time. Moreover, the samples from both groups were handled similarly. A future RCT may resolve this oxytocin level difference. *Second*, the number of participants were limited. Based on the *p*-values before Bonferroni correction in Table 3, increasing the sample size may increase the power. However, considering that early-term births (before 39 weeks of gestation) are associated with adverse neonatal outcomes [31, 32], the timing of the intervention cannot be carried out any earlier than what was implemented in the present study.

The strength of this research is the unified intervention under a strict supervision. Previous RCTs of breast stimulation were not strictly controlled interventions because they were mainly carried out at home. It is valuable to find a significant difference in the 2 oxytocin groups under this setting.

In future RCTs, a large sample size and measurement of the Bishop score are warranted to fully clarify the effects of breast stimulation on the oxytocin level in relation to labor induction, onset, and augmentation. In addition, it was suggested that repeated stimulations may increase the oxytocin level rather than a longer stimulation at one time as shown in this study. Also, it is desirable to perform a comparative study in which the rate of spontaneous labor onset is set as the primary outcome, as well as to set up multiple intervention groups that perform long and short periods (30 min or less) of breast stimulation.

## Conclusion

Breast stimulation for 1 h each day for 3 days resulted in a significantly higher estimated change in the mean oxytocin level at 30 min postintervention on the third day in the intervention group than in the control group. Repeated breast stimulations for spontaneous onset of labor caused an increase in the salivary oxytocin level. Further studies are required wherein the rate of spontaneous labor onset is set as the primary outcome from multiple intervention groups to clearly identify an effective breast stimulation method.

## Data Availability

All raw data generated or analyzed during this study are available from the corresponding author upon reasonable request.

## Abbreviations

ANCOVA: Analysis of Covariance
FHR: Fetal Heart Rate
RCT: Randomized Control Trial
SNP: Single Nucleotide Polymorphism
